# TRAP1 functions in the morphogenesis of the embryonic kidney

**DOI:** 10.1080/19768354.2025.2477789

**Published:** 2025-03-12

**Authors:** Ha Eun Kim, Taejoon Kwon, Hyo Jung Sim, Tae Joo Park

**Affiliations:** a Department of Biological Sciences, College of Infaormation-Bio Convergence Engineering, Ulsan National Institute of Science and Technology, Ulsan, Republic of Korea; b Department of Biomedical Engineering, College of Information-Bio Convergence Engineering, Ulsan National Institute of Science and Technology, Ulsan, Republic of Korea; c Center for Genomic Integrity, Institute for Basic Science, Ulsan, Republic of Korea

**Keywords:** TRAP1, kidney, muscle, Xenopus

## Abstract

TNF receptor-associated protein1 (TRAP1) is a mitochondrial molecular chaperon with high homology with a cytosolic chaperon HSP90. It has been shown that TRAP1 functions as an inhibitor for apoptosis by preventing cytochrome-c release from mitochondria. In addition, TRAP1 seems to play critical roles in metabolic processes for energy production, such as glycolysis and β-oxidation. It has also been reported that TRAP1 is a direct target of PTEN-induced kinase 1 (PINK1) and may be a cause of Parkinson’s disease (PD) in humans. Although the biochemical functions of TRAP1 are under intense study for the physiology and treatment of various cancers, its roles in vertebrate development have not been reported. This study shows that *Xenopus* TRAP1 is strongly expressed in the developing muscle, kidney, and brain tissues. Perturbation of TRAP1 function by treating TRAP1 inhibiter GTPP or microinjection of antisense-morpholino oligo (MO) caused mild defects in striated muscle fiber formation. Furthermore, the looping patterns of developing kidney tubules were perturbed, indicating that TRAP1 function is necessary for proper kidney development.

## 1. Introduction

TNF receptor-associated protein 1 (TRAP1) is a mitochondrial molecular chaperone with high homology with the cytosolic chaperone HSP90. TRAP1 has been implicated in various cellular processes, including apoptosis inhibition, metabolic regulation, and mitochondrial function maintenance (Joshi et al. 2022). While TRAP1’s biochemical functions have been extensively studied in the context of cancer and neurodegenerative diseases, its roles in vertebrate development remain largely unexplored.

TRAP1 is an apoptosis inhibitor that prevents cytochrome C release from mitochondria (Cechetto and Gupta 2000; Joshi et al. 2022). Additionally, it plays critical roles in metabolic processes for energy production, such as glycolysis and β-oxidation (Joshi et al. 2022). Recent studies have also identified TRAP1 as a direct target of PTEN-induced kinase 1 (PINK1), suggesting a potential link to Parkinson’s disease (PD) pathogenesis (Zhang et al. 2013).

The importance of proper protein folding and mitochondrial function for normal tissue formation in developmental biology has been well established. However, the specific role of TRAP1 in these developmental processes has not been previously reported. *Xenopus laevis* is a favorable model system for studying gene functions in organ and tissue development (Umair et al. 2020; Kim et al. 2024a), and its oocyte extract has been widely used to study DNA damage responses (Kim et al. 2024b). In this study, we aim to address this knowledge gap by investigating the expression and function of TRAP1 during embryonic development using *Xenopus laevis*. We focused on several key aspects of TRAP1’s role in development, including its expression pattern in developing *Xenopus* tissues, the effects of TRAP1 inhibition on skeletal muscle and cartilage development, the impact of TRAP1 knockdown on muscle and cartilage formation, and its role in kidney development, particularly in tubule formation.

Our findings reveal that *Xenopus* TRAP1 is strongly expressed in developing skeletal muscles and kidneys. Interestingly, treatment with the TRAP1 inhibitor GTPP caused severe defects in craniofacial cartilage and muscles. However, knocking down TRAP1 using antisense morpholino oligonucleotides (MO) resulted in less severe effects on skeletal muscles and cartilage. This discrepancy suggests that TRAP1 may have both chaperone-dependent and independent functions during development or that compensatory mechanisms may be activated upon genetic knockdown but not pharmacological inhibition.

Furthermore, TRAP1 function is necessary for adequately looping kidney tubules, indicating an essential role in kidney development. This finding expands our understanding of TRAP1’s role beyond its known functions in cancer and neurodegeneration, highlighting its importance in organogenesis.

These results provide novel insights into the developmental functions of TRAP1, expanding our understanding of this critical chaperone beyond its well-established roles in disease contexts. Our study lays the groundwork for future investigations into the mechanisms by which TRAP1 influences tissue development, particularly in skeletal muscles, cartilage, and kidneys. These findings may affect our understanding of developmental disorders and potential therapeutic approaches targeting TRAP1 function.

## 2. Materials and methods

### 2.1. Prepare of *Xenopus* embryo

An adult female *Xenopus laevis* was ovulated by injecting human chorionic gonadotropin (HCG), and the eggs were fertilized in vitro. The jelly layer was removed by swirling the eggs in 3% L-cysteine, pH 7.9 (Sigma), in 1/3X MMR (Marc’s Modified Ringers) solution. Fertilized eggs were kept in 1/3X MMR until the two-cell stage for microinjection. The adult *Xenopus* laevis are provided by the Korea National Research Resource Center (KNRRC, KXRCR000001, KXRCR000002).

### 2.2. Microinjection

Embryos were placed in a 3% ficoll in 1/3x MMR for microinjections. They were injected into the dorsal-vegetal region of each blastomere at the two-cell stage using Picospritzer III (Parker) and MK1 microinjector (Singer instrument). The injected embryos were grown in 1/3X MMR with gentamycin. We designed splicing-blocking antisense morpholinos for TRAP1 from Gene Tools; (TRAP1 Int MO: CTCAATAGGCAAGGACCTACCTCTT). For experiments, 40ng-80 ng of morpholinos and various amounts of mRNAs were used, and 40 ng of morpholinos were mainly injected for phenotype analysis.

### 2.3. Cloning

*Xenopus* TRAP1 cDNA was cloned from total RNA extracts at stage 35 embryos. Total RNA was purified from embryos and reverse-transcribed with random hexamer primer (Life Technologies) and Reverse Transcriptase (Promega). *Xenopus* TRAP1 cDNA was amplified using primers; forward: 5‘-aattgaattcTGGGTTCCAGCGTGTCTCTTTAC-3’, Backward: 5‘-aattctcgagCCATTCCTCATCCAAGCCATAAG-3’. The amplified PCR product is cloned into the CS108 vector by T4 DNA ligase (NEB, New England Biolabs). TRAP1 mRNAs were prepared using an mMessage machine (Ambion) according to the manufacturer’s manual. The TRAP1 probe is synthesized using T7 RNA polymerase (NEB).

### 2.4. Whole-mount in situ hybridization

The whole-mount in situ hybridization was performed as described previously (Moorman et al. 2001).

Dr. Richard Harland at the University of California, Berkeley, provided the cDNA constructs for antisense probe synthesis.

### 2.5. Cartilage staining

Embryos were fixed in 1X MEMFA (1x MEM salt, 4% formaldehyde) and dehydrated in 100% ethanol. The embryos were rehydrated by serial washing in 90%, 75%, 50%, and 25% ethanol for 10 minutes. The embryos were then stained in the Alcian blue staining solution (80% ethanol, 10 mM Magnesium Chloride, 0.04% Alcian Blue) for 24 hr. After washing two times in washing solution (80% ethanol, 10 mM Magnesium Chloride), embryos were bleached. The embryos were cleared in trypsin solution. The stained embryo is imaged using a stereomicroscope (Olympus SZX16).

### 2.6. Immunoblotting

For western blot analysis, control and mutant embryos were homogenized in ice-cold lysis buffer (50 mM Tris pH 7.4, 105 mM NaCl, 0.1% Triton X-100, 5% Glycerol), with protease inhibitor (Thermo). Homogenated samples were centrifuged at 13,200 rpm for 15 minutes at 4℃. Proteins were blotted to 8% polyacrylamide gel. The protein blots were performed with anti-TRAP1 antibody (BD Bioscience) and anti-α-tubulin (Abcam) for loading control. Data was collected using ImageQuant LAS 4000 (GE Healthcare Life Science).

### 2.7. Immunofluorescence and microscopy

Embryos were fixed in 1x MEMFA (1x MEM salt, 4% formaldehyde), and fixed embryos were washed with PBS. Then, the embryos were serially washed in 5, 10, and 15% sucrose for 2 h. The embryos were embedded in an Optimal Cutting Temperature (OCT) compound (Sakura Finetek) and were frozen in a −80 ℃ freezer. The frozen samples were sectioned in 10 μm thicknesses using a cryotome (Thermo Scientific, HM56O).

Sectioned slices were incubated in blocking solution (10% FBS, 2% DMSO, 0.1% Triton X-100 in TBS) at room temperature for 30 minutes, and primary antibodies and secondary antibodies were incubated for 1hr after washing in TBST (0.1% Triton X-100 in TBS). Immunostaining was performed with the following antibodies: anti-MHC (DSHB 12/101), anti-TRAP1 (BD Bioscience), anti-V5 (Santa Cruz Biotechnology), anti-WGA (Molecular Probes), anti-DAPI (Sigma) for primary; Quantitative analysis of kidney tubule area was measured using Origin9 F-test.

### 2.8. TUNEL staining

TUNEL staining is performed with in Situ Cell Death Detection Kit (Roche) according to the manufacturer’s manual. Sectioned samples were permeabilized in citrate buffer (0.1% Triton X-100, 0.1% sodium citrate) for 5 minutes on ice. TUNEL staining was performed for 2 hours at 37℃. The stained sample is imaged using a confocal microscope (Zeiss LSM700) or stereomicroscope (Olympus SZX16). Image analysis was performed using the ZEN program (Zeiss).

## 3. Result

### 3.1. TRAP1 is expressed in developing skeletal muscles and kidneys

**Figure 1.**
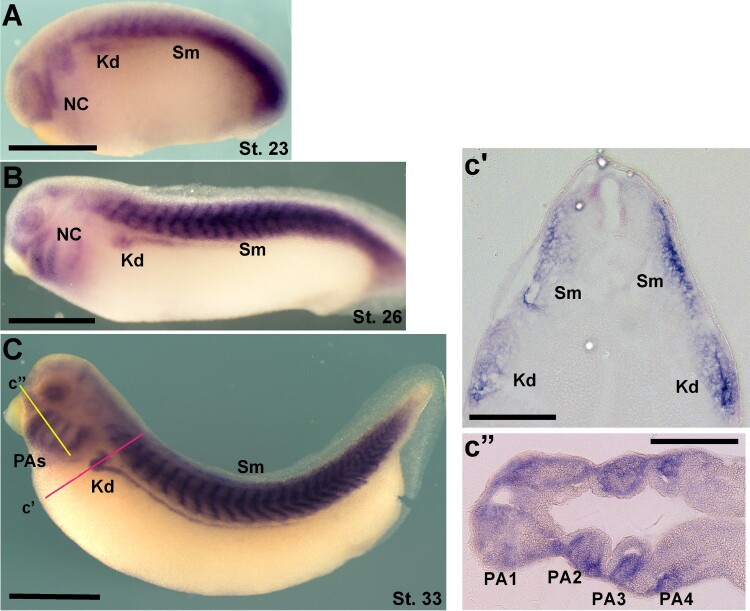
TRAP1 is expressed at developing neural crest cells, somite, kidney, and pharyngeal arches. TRAP1 expression in Xenopus embryos was analyzed by performing an RNA whole-mount in situ hybridization (WISH) assay. (A) TRAP1 is predominantly expressed in the presomitic tissues and neural crest cells. Scale bar = 500 μm. (B) At early tailbud stages, TRAP1 is expressed at somite and pharyngeal arches. Scale bar = 500 μm. (C) At late tailbud stages, TRAP1 is expressed at somite, pharyngeal arches, and kidney Scale bar = 500 μm. c′, c″. Expression pattern of TRAP1 in sectioned embryos. The sectioning plane is indicated in the C as yellow and red lines. Scale bar = 200 μm. NC; Neural crest cells, Kd; Kidney, Sm; Somite, PAs; Pharyngeal arches.

To assess the functions of TRAP1 in the embryonic development of vertebrates, we first systematically analyzed the expression patterns of TRAP1 in *Xenopus* embryos by performing an RNA whole-mount in situ hybridization (WISH) assay. WISH analysis showed TRAP1 is strongly expressed in developing somites, kidney, and cranial neural crest cells. At the early neurula stage, TRAP1 is predominantly expressed in the presomitic tissues and developing somites (Figure 1A). We also observed weak expression of TRAP1 in migrating cranial neural crest cells and presumptive kidney tissues (Figure 1A, B). At tailbud stages, TRAP1 expression is expanded to the entire somite areas and pharyngeal arches (Figure 1C, C″). Also, TRAP1 expression in the kidney is markedly enhanced (Figure 1C, C′). The dynamic expression of TRAP1 in developing embryos suggests that TRAP1 could be involved in the development of multiple tissues and organs in vertebrates.

### 3.2. TRAP1 inhibitor GTPP causes severe defects in craniofacial and cartilage and muscles

The biochemical function of TRAP1 is at the center of cancer metabolism (Matassa et al. 2018; Wengert et al. 2022), and the TRAP1 inhibitor was extensively examined and is currently used by many researchers (Kang et al. 2007). One well-characterized TRAP1 inhibitor is Gamitrinip-TPP (GTPP), an HSP70 inhibitor with a mitochondrial targeting moiety (Kang et al. 2007). Therefore, we exploited the TRAP1 inhibitor GTPP to study the loss-of-function phenotype of TRAP1 in developing embryos.

**Figure 2.**
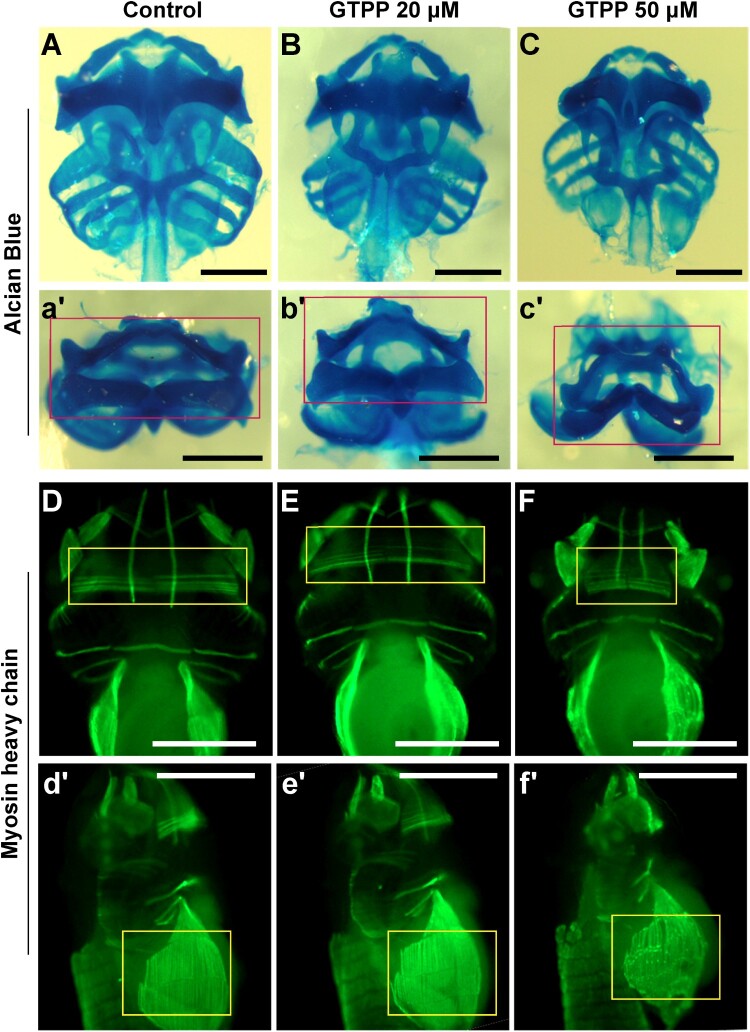
TRAP1 inhibition causes severe defects in craniofacial and cartilage and muscles. The embryos were grown in various concentrations of TRAP1 inhibitor, GTPP, until Stage 45, and the embryonic phenotypes were analyzed. (A–C) The craniofacial cartilages were analyzed using alcian blue staining. 20μM of GTPP did not cause any noticeable phenotypes. 50μM of GTPP severely disrupted normal facial morphogenesis (red rectangles). Scale bar = 500 μm. (D–F) The embryonic muscles were analyzed by immunostaining muscle myosin heavy chain. The interhyoideus and body wall muscles were unaffected at the embryos in 20μM of GTPP. However, in 50μM of GTPP, embryos were significantly affected and noticeably hypomorphic compared to control embryos. Scale bar = 500 μm.

The embryos were grown in various concentrations of GTPP until Stage 45 when most of the major organs were fully functional. 20μM of GTPP did not cause any noticeable phenotypes; however, 50μM of GTPP severely disrupted normal facial morphogenesis. To assess the underlying cause of the facial defects, we analyzed facial muscles and cartilage by immunostaining and alcian blue staining, respectively. Alcian blue staining of – treated embryos displayed medially bent facial cartilages (Figure 2A–C); however, the chondrogenesis and patterning of facial cartilages did not seem to be affected. We also analyzed the muscles in GTPP-treated embryos. The embryos grown in the presence of 50μM of GTPP displayed severe hypomorphic muscles. The interhyoideus and body wall muscles were significantly affected and noticeably hypomorphic compared to control embryos (Figure 2D–F). Given the strong expression of TRAP1 in somitic muscles and pharyngeal arches, the hypomorphic muscles in GTPP-treated embryos suggest that TRAP1 function is necessary for proper muscle development. Additionally, GTPP-treated embryos showed a tail-bending phenotype supporting this suggestion (Sup. 1).

### 3.3. TRAP1 knockdown did not cause severe damage to skeletal muscles and cartilage

**Figure 3.**
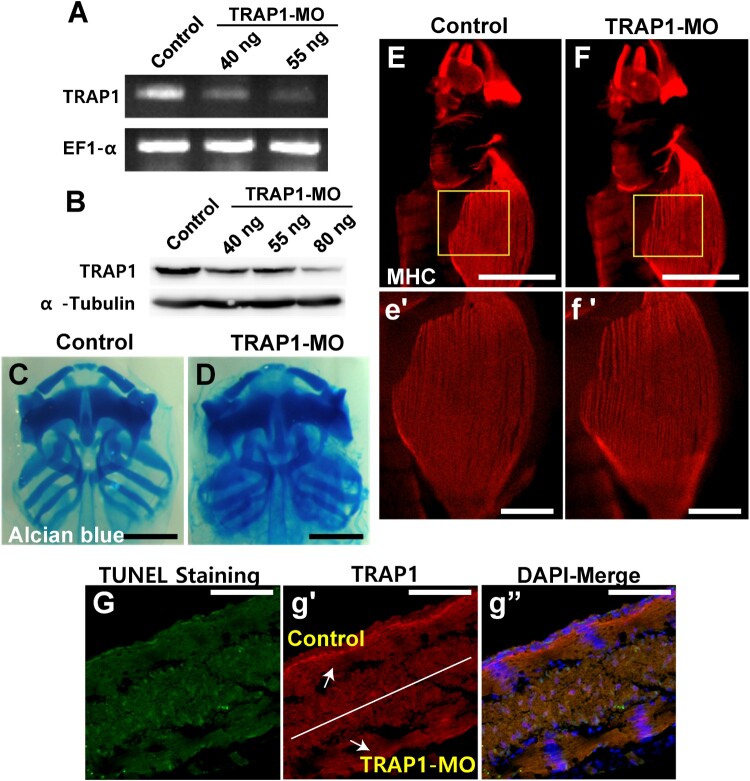
TRAP1 knockdown did not cause severe damage to skeletal muscles and cartilage. TRAP1 expression was depleted by micro-injection of the antisense morpholino oligo (MO). (A–B) RT-PCR and western blot analysis showed that the antisense MO injection efficiently decreased TRAP1 expression. (C–D) The craniofacial cartilages were analyzed using alcian blue staining. TRAP1 knockdown did not severely disrupt cartilage development. Scale bar = 500 μm. (E–F) The embryonic muscles were analyzed by immunostaining muscle myosin heavy chain. The areas highlighted by yellow rectangles were displayed in e’ and f’ with higher magnification. We observed mild defects in body wall muscles. Scale bar = 500 μm. (G) TRAP1-MO was injected into half of the embryos by Unilateral injection. The TRAP1-MO efficiently blocked the expression of TRAP1 in the targeted sites of the embryos (g’), but apoptotic cell death was not increased. Scale bar = 100 μm.

Although the TRAP1 inhibitor GTPP is known to be efficiently targeted to the mitochondria and inhibit TRAP1, we next tested if the knockdown of endogenous TRAP1 causes identical phenotypes as those of GTPP-treated embryos. We specifically knockdowned TRAP1 expression by micro-injection of the antisense morpholino oligo at two-cell stage embryos. Semi-quantitative RT–PCR and western blot analysis showed that the antisense MO injection efficiently decreased TRAP1 expression in developing embryos (Figure 3A,B). Next, we analyzed craniofacial cartilage and muscle development in TRAP1 knockdowned embryos. However, TRAP1 knockdown did not severely disrupt cartilage development (Figure 3C,D). We only observed mild defects in body wall muscles (Figure 3E,F). In drosophila, TRAP1 knockdown caused increased cell death in muscle cells, resulting in lower muscle fibers (Costa et al. 2013). Therefore, we examined if TRAP1 knockdown increased apoptotic cell death in the muscles. To compare TRAP1 knockdown muscle tissues and wild-type tissue more efficiently, we targeted the antisense morpholino into half of the embryos by injecting it into one blastomere at two-cell stage embryos. Unilateral injection of the TRAP1-MO efficiently blocked the expression of TRAP1 in the targeted sites of the embryos (Figure 3g′). However, the knockdown of TRAP1 did not severely increase apoptotic cell death in the somitic muscles compared to the non-injected somitic muscles (Figure 3G). This discrepancy between TRAP1 inhibitor treatment and MO-mediated knockdown may be because GTPP can inhibit TRAP1 function much more efficiently. Although TRAP1-MO injection efficiently knockdowned the expression level of TRAP1, the residual activity of TRAP1 can still exist.

### 3.4. TRAP1 function is necessary for the proper looping of kidney tubules

**Figure 4.**
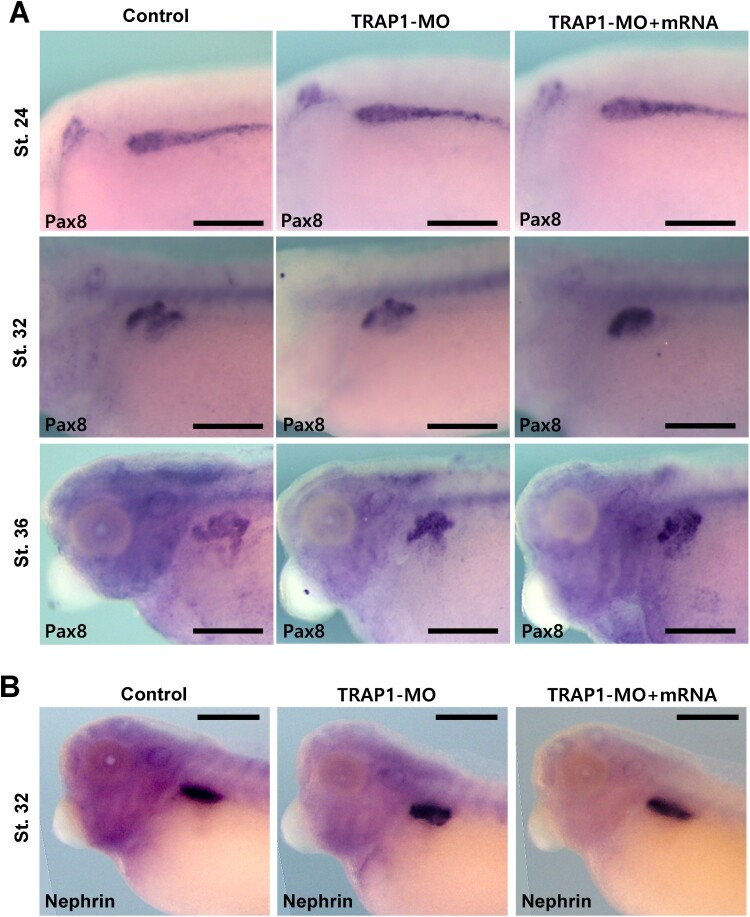
TRAP1 knockdown did not affect the expression of early kidney genes. Pax8 and Nephrin expression were analyzed by WISH during kidney development. (A, B) TRAP1 knockdown did not significantly change the expression of Pax8 and Nephrin in developing kidneys. Scale bar = 200 μm.

**Figure 5.**
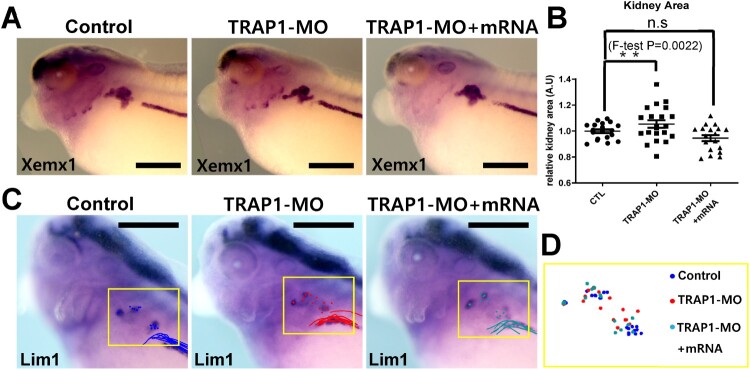
TRAP1 function is necessary for the proper looping of kidney tubules. Xemx1 and Lim1 expression were analyzed by WISH to visualize kidney tubules. (A, B) The area of Xemx1 expression in proximal kidney tubules was measured, and the Gaussian distribution of kidney areas was analyzed by F-test. The average of the kidney area was not distinguishable. However, in TRAP1 morphants, the distribution of the value of the total kidney tubule area was considerably broader than that of control embryos. Scale bar = 200 μm. (CTL; n = 19, TRAP1-MO; n = 21, TRAP1-MO + mRNA; n = 18) (C) The expression of Lim1 in developing kidneys was analyzed by WISH, and the Lim1 signals of embryos were overlayed according to the first anterior-most nephrostome and distal kidney tubules. Scale bar = 200 μm. (D) Image of overlayed Lim1 positive nephrostomes.

Recent research has reported that TRAP1 may be a causative gene for congenital abnormalities of the kidney and urinary tract (CAKUT) (Saisawat et al. 2014). Since TRAP1 is strongly expressed in developing kidneys, we analyzed the kidney phenotypes in TRAP1 knockdowned embryos. To this end, we performed a WISH analysis using several well-known markers in kidney development. Pax8 is a critical transcription factor for the earliest steps of pronephric development. However, we could not observe any noticeable changes in gene expression patterns in developing kidneys in the TRAP1 knock-downed embryos (Figure 4). Based on the Pax8 expression (Buisson et al. 2015), the early kidney specification and differentiation were typically achieved (Figure 4A). Also, the Nepherin expression which is more specifically related to the later stages in pronephric glomus (Gerth et al. 2005) did not change significantly in the morphant embryos (Figure 4B). Other kidney markers such as Xemx1 and Lim1 (Brandli 1999; Chan et al. 2000) were also expressed normally during pronephric kidney differentiation to distinct kidney tubules such as proximal and distal tubules (Figure 5A,C). Then, we further measured the area of proximal tubules after Xemx1 WISH and compared the differences in the proximal tubule area between the control and TRAP1 morphant. The average proximal tubule areas in control and TRAP1 morphants were not significantly different. However, to our surprise, the Gaussian distribution of kidney areas in the TRAP1 morphant was considerably broader than that of control embryos (Figure 5B). Then, we analyzed how far the data were scattered from the mean in control and TRAP1 morphant embryos by performing the F-test analysis (Figure 5B). Indeed, the distribution of the data in the TRAP1 morphants is significantly different from those of the control. Further, the Gaussian distribution of Xemx1-marked kidney tubule areas was recovered by co-injection of TRAP1-MO and TRAP1 mRNA. We further analyzed the overall positions of nephrostomes and distal kidney tubules by visualizing Lim1 expression (Figure 5C). Nephrotomes in developing *Xenopus* pronephros are the opening of proximal kidney tubules that moves blood filtrate from the glomus into the proximal tubules (Blackburn and Miller 2019) which can indicate the overall structure of pronephros. Then, the Lim1 expression patterns of dozens of embryos were overlayed according to the first anterior-most nephrostome and distal kidney tubules (Figure 5D). The overlayed images indicated that the overall shape of the kidney tubules in the TRAP1 morphant embryos was significantly variable compared to the control and rescued embryos.

These data indicate that the knockdown of TRAP1 expression affects the typical looping pattern of the kidney proximal tubule.

## 4. Discussion

This study provides novel insights into the developmental functions of TRAP1 in *Xenopus* embryos, expanding our understanding of this vital chaperone beyond its well-established roles in cancer and neurodegenerative diseases. Our findings demonstrate that TRAP1 plays a crucial role in developing skeletal muscles, cartilage, and kidneys during *Xenopus* embryogenesis.

The strong expression of TRAP1 in developing somites, kidneys, and cranial neural crest cells suggests its involvement in the formation and function of these tissues (Figure 1). This tissue-specific expression pattern aligns with the observed developmental defects upon TRAP1 inhibition or knockdown, particularly in muscle and kidney development.

Interestingly, we observed a notable difference in phenotypes between TRAP1 inhibition using GTPP and genetic knockdown using morpholinos. GTPP treatment resulted in severe defects in craniofacial cartilage and muscle development, while morpholino-mediated knockdown produced milder effects (Figures 2, 3). This difference could be attributed to several factors, including the existence of both chaperone-dependent and independent functions of TRAP1, activation of compensatory mechanisms in response to genetic knockdown, or differences in the efficiency of pharmacological inhibition versus genetic knockdown. Further investigation is needed to elucidate the precise reasons for these differences and better understand the full spectrum of TRAP1 functions during development.

Also, there is a possibility that there may be other reasons for this. GTPP is a mitochondria-targeted Hsp90 inhibitor designed to accumulate in the mitochondrial matrix (Fiesel et al. 2017; Xie et al. 2021). It inhibits the ATPase activity of both mitochondrial HSP90 and TRAP1, accumulating unfolded proteins and subsequently activating the mitochondrial unfolded protein response (mtUPR) (Criscuolo et al. 2021). This broader inhibition could contribute to some of the observed effects, rather than solely due to TRAP1-specific inhibition.

Our finding that TRAP1 knockdown affects the looping patterns of developing kidney tubules is particularly intriguing (Figure 5). This observation aligns with recent reports suggesting TRAP1 as a potential causative gene for congenital abnormalities of the kidney and urinary tract (CAKUT). The molecular mechanisms by which TRAP1 regulates proper tubule morphogenesis remain to be elucidated and represent an important area for future research.

In conclusion, our study reveals novel developmental functions of TRAP1 in *Xenopus*, particularly in skeletal muscle, cartilage, and kidney formation. These findings expand our understanding of TRAP1 beyond its known roles in cancer and neurodegeneration, highlighting its importance in organogenesis. Future research should focus on elucidating the molecular mechanisms by which TRAP1 influences tissue development and exploring its potential as a therapeutic target for developmental disorders.

## Ethical approval statement

All animal experiments were performed with appropriate ethical approval from the UNIST Institutional Animal Care and Use Committee (UNISTIACUC-22-60).

## Supplementary Material

Supplementary Material

## Data Availability

All materials and data newly created in this work are available upon request.
