# Supplementary material for: TRAP1 functions in the morphogenesis of the embryonic kidney
Source: Anim Cells Syst (Seoul). 2025 Mar 12;29(1):9–18. doi: 10.1080/19768354.2025.2477789 (PMC11912273; doi:10.1080/19768354.2025.2477789)
Supplement: Supplementary Material [file TACS_A_2477789_SM9197.docx]

**Supplementary information**

**CTL**

**GTPP 20 μM**

**GTPP 50 μM**

**Supplementary figure 1.**

The brightfield images of the embryos grown in various concentrations of TRAP1 inhibitor, GTPP. The embryo grown in 50 μM of GTPP showed tail-bending phenotypes.
